# Challenge of Pigs with Classical Swine Fever Viruses after C-Strain Vaccination Reveals Remarkably Rapid Protection and Insights into Early Immunity

**DOI:** 10.1371/journal.pone.0029310

**Published:** 2012-01-03

**Authors:** Simon P. Graham, Helen E. Everett, Felicity J. Haines, Helen L. Johns, Olubukola A. Sosan, Francisco J. Salguero, Derek J. Clifford, Falko Steinbach, Trevor W. Drew, Helen R. Crooke

**Affiliations:** 1 Virology Department, Animal Health and Veterinary Laboratories Agency, Addlestone, United Kingdom; 2 Pathology and Host Susceptibility Department, Animal Health and Veterinary Laboratories Agency, Addlestone, United Kingdom; 3 Specialist Scientific Services, Animal Health and Veterinary Laboratories Agency, Addlestone, United Kingdom; Nanyang Technological University, Singapore

## Abstract

Pre-emptive culling is becoming increasingly questioned as a means of controlling animal diseases, including classical swine fever (CSF). This has prompted discussions on the use of emergency vaccination to control future CSF outbreaks in domestic pigs. Despite a long history of safe use in endemic areas, there is a paucity of data on aspects important to emergency strategies, such as how rapidly CSFV vaccines would protect against transmission, and if this protection is equivalent for all viral genotypes, including highly divergent genotype 3 strains. To evaluate these questions, pigs were vaccinated with the Riemser® C-strain vaccine at 1, 3 and 5 days prior to challenge with genotype 2.1 and 3.3 challenge strains. The vaccine provided equivalent protection against clinical disease caused by for the two challenge strains and, as expected, protection was complete at 5 days post-vaccination. Substantial protection was achieved after 3 days, which was sufficient to prevent transmission of the 3.3 strain to animals in direct contact. Even by one day post-vaccination approximately half the animals were partially protected, and were able to control the infection, indicating that a reduction of the infectious potential is achieved very rapidly after vaccination. There was a close temporal correlation between T cell IFN-γ responses and protection. Interestingly, compared to responses of animals challenged 5 days after vaccination, challenge of animals 3 or 1 days post-vaccination resulted in impaired vaccine-induced T cell responses. This, together with the failure to detect a T cell IFN-γ response in unprotected and unvaccinated animals, indicates that virulent CSFV can inhibit the potent antiviral host defences primed by C-strain in the early period post vaccination.

## Introduction

A number of live attenuated classical swine fever (CSF) vaccines have been developed by traditional methods such as passage in tissue culture (e.g. Thiverval strain), or by serial passage in rabbits (e.g. the Chinese or C-strain) [1]. These vaccines are remarkably effective in abrogating the clinical signs of disease and are used to control losses in areas where the disease is endemic [2]. Combined with culling of infected animals, such vaccines have assisted in the eradication of the disease from many areas of the world, and are being applied to eliminate disease from wildlife reservoirs [3], [4], [5]. However, due to the inability to identify infected animals by serology within a vaccinated population, the non-emergency use of such live attenuated vaccines is banned in the EU [6] and current strategies to control outbreaks are based on slaughter of all pigs on infected holdings, establishment of protection and surveillance zones, movement restrictions and, in some instances, pre-emptive culling of neighbouring uninfected herds [3], [6]. This policy can lead to large scale economic and social consequences, which were most strikingly demonstrated by the devastating outbreak that occurred in the Netherlands, France, Belgium and Spain in 1997 [7], [8]. A much smaller outbreak in the UK in 2001 resulted in the destruction of around 73,000 animals, 32,000 of which were in herds pre-emptively slaughtered because they were considered as dangerous contacts, although they were not subsequently identified to be infected [9]. In the last decades the morality of culling healthy animals for economic interests has been questioned, despite the fact that food production animals are destined for slaughter anyway [10]. This has led to a reconsideration of options for disease control, including the use of emergency vaccination. Decisions on control strategies are complex, will vary for different regions, and depend on many factors, such as; herd density, production system(s), the presence of susceptible wildlife, impact on export trade and current opinions on economic versus ethical factors [11], [12].

Like pre-emptive culling, the aim of an emergency vaccination strategy is to reduce virus spread from an infected herd. With current legislation and available CSF vaccines, possible emergency vaccination strategies include: 1) suppressive vaccination, where animals in a zone around an identified premises are vaccinated with live-attenuated vaccine and subsequently slaughtered (vaccinate-to-kill) or 2) protective vaccination, (vaccinate to live) where a marker vaccine that allows identification of infection within a vaccinated population is used. Unlike suppressive vaccination, animals vaccinated under a protective strategy, and their products, would be able to enter normal trade, [6], [13], [14]. The currently licensed marker vaccine is not ideal in terms of efficacy, and the accompanying differential test is not optimal for a rapid emergency vaccination strategy [13], [15], [16], [17] and many research efforts are aimed at developing improved marker vaccines and diagnostics [18], [19]. To date, emergency vaccination strategies have not been used in an outbreak, apart from transitionally in Romania, and so conclusions about how well they may perform are largely reliant on simulations [12], [20].

Although live attenuated vaccines have been used prophylatically for many years, some questions remain about how effective they would be at controlling CSF in a suppressive emergency vaccination campaign. To prevent infection of neighbouring herds, suppressive vaccination should induce a rapid protection that prevents dissemination of virus. The C-strain vaccine provides solid protection against challenge by 7 days post-vaccination (dpv) [1] and there are indications that protection occurs even earlier [21], [22], [23]. Dewulf and colleagues [24] demonstrated that vaccination with C-strain on the same day as challenge prevented transmission to vaccinated pen mates. However, fewer data are available on how rapidly vaccination prevents transmission of virus to susceptible animals in these very short intervals.

For CSF-free areas, introductions of CSFV could originate from any region of the world and so an emergency vaccine should provide protection against all CSFV strains. C-strain, and the majority of live attenuated CSF vaccines, belong to genotype 1.1 and are considered to be effective against all genotypes. Experimental studies have demonstrated vaccine efficacy against for genotype 1 [25], [26] and genotype 2 strains [24], [27]. However, there is evidence that genotype 2 strains have replaced genotype 1 viruses as the dominant genotype in China, an area where vaccination is mandatory, implying that antigenic differences between genotypes may impact to some extent on vaccine efficacy [28], [29]. In addition, very little information is available on vaccine efficacy against viruses of the genotype 3 subgroups. Parchariyanon and co-workers [30] described 18 genetically diverse genotype 3.3 isolates, two of which, CBR/94/2 and CBR/93, have a distinct antigenic profile and which, notably, only react poorly with Mab WH303 which targets a principal neutralising epitope TAVSPTTLRP of the viral E2 envelope protein [31]. The ability of C-strain to protect against clinical signs upon challenge with CBR/94/2 two weeks after vaccination has been demonstrated [32]. However, the mechanism of protection has not been elucidated and it is unclear if C-strain vaccine protects efficiently against transmission of genetically and antigenically diverse strains at very short periods post-vaccination.

It is generally accepted that neutralising antibody induced by vaccination with C-strain, which is detected from 2–3 weeks post-vaccination [33], is a major protective mechanism. However, for a rapid emergency vaccine, the mechanism of protection induced prior to this time is of greater importance. Since virus-specific T cell IFN-γ responses can be detected 7 days post-vaccination it has been suggested that they may mediate protection in the absence of antibody [27], [34]. C-strain-induced T cell responses have been reported in a limited number of studies. Virus-specific CD4^+^ and CD8^+^ T cell IFN-γ responses, targeting the major viral envelope glycoprotein E2 and the non-structural viral protein NS3, have been observed following vaccination [35], [36], [37]. MHC class I restricted cytotoxic T lymphocyte responses, directed against epitopes on both E2 and NS3 have also been described, [35], [36], [38]. We have recently shown that immunisation of pigs with C-strain vaccine induced a robust, virus-specific, IFN-γ response detectable from at least 9 days post-vaccination but these responses could not be detected in pigs experimentally infected with a recent, moderately virulent, field isolate [39]. While IFN-γ appears to serve as a marker for anti-CSFV cell-mediated responses, the data underlying a correlation with protection are limited.

With a view to providing additional information for those formulating emergency vaccination policies and to understand the role that T cell responses may have in vaccine-induced, rapid immunity we have investigated the speed with which the C-strain vaccine affords protection of susceptible in-contact animals, after challenge with two diverse CSFV strains, as well as the kinetics and magnitude of T cell responses.

## Materials and Methods

### Ethics statement

The project was approved by the Animal Health and Veterinary Laboratories Agency ethics committee and all procedures were conducted in accordance with the UK Animals (Scientific Procedures) Act 1986 under project licence permit number PPL 70_6559).

### Animals and Viruses

Large White/Landrace cross male pigs, 9 weeks of age were purchased from a local commercial source. During the acclimatisation period, biothermal microchips (Destron Technologies Inc.) were implanted subcutaneously behind the left ear for temperature monitoring. CSFV strain UK2000/7.1 [40] is a genotype 2.1 virus isolated from a domestic pig during an outbreak in the UK in 2000. CBR/93 was kindly provided by S. Parchariyanon [30]. Viruses were propagated in the PK-15 swine kidney cell line [41]. Lyophilized live attenuated Riemser® C-strain CSFV vaccine (AC Riemser Schweinepestvakzine, Riemser Arzneimittel AG, Germany) was reconstituted with the supplied buffer as directed, immediately prior to vaccination.

### Challenge experiments

The protection afforded by vaccination against challenge with the UK2000/7.1 and CBR/93 strains was assessed in two independent challenge experiments. In both experiments three groups of 6 pigs were vaccinated intramuscularly with 2 ml of reconstituted vaccine, either 5, 3 or 1 day prior to challenge. Three additional unvaccinated pigs were housed with each group of vaccinated animals in order to monitor prevention of infection of in-contact animals. A fourth group, consisting of 6 animals, remained unvaccinated. Four of these animals were challenged whilst the remaining two were not and served to monitor the extent of in-contact infection. On day 0 animals to be challenged were moved to other rooms and challenged with an intranasal aerosol using a MAD 300 device (Wolfe Tory Medical, USA). Titration of the inoculae indicated challenge doses were 10^4.6^ TCID_50_ for UK2000/7.1 and 10^5.1^ TCID_50_ for CBR/93. After 24 hours, the challenged animals were re-introduced to the in-contact animals in the original rooms.

Throughout the duration of the study, temperatures and clinical signs were recorded twice daily, using a slightly modified scoring scheme as described previously [42], [43]. EDTA blood and serum samples were obtained prior to vaccination and then at 2 to 3 day intervals. Nasal swabs were collected at 1–3 day intervals. Animals were euthanized by IM administration of Ketamine/Rompun sedative followed by IV administration of 20% sodium pentobarbitone solution.

### Virus quantification in blood and nasal swabs, haematology and pathology

Nasal swabs were soaked in 1 ml PBS, agitated and then centrifuged at 524 g for 7 min. Blood samples were collected in EDTA vacutainers (BD Biosciences). Viral RNA was extracted from 140 µl nasal swab suspension or 50 µl EDTA blood using a Qiagen BioRobot Universal instrument. For swabs, a QIAamp 96 DNA Swab BioRobot kit (Qiagen) was used. For blood, the All-for-One Nucleic acid kit (Qiagen) was used with a QIAamp One For All UNIV rcV72 programme, which includes a proteinase K digestion step. Viral RNA was quantified using a qRT-PCR one-step Superscript III Platinum kit (Invitrogen) as described previously [43]. Platelets and CD 45^+^ white blood cells present in EDTA blood were quantified by flow cytometry [43]. Post mortem examinations followed standard operational procedures, and any observed lesions were recorded. Tissue samples were fixed in buffered formalin and routinely processed into paraffin wax. Tissue sections were stained with haematoxylin and eosin for histopathological analysis. Macropathology and histopathological scoring systems were used as previously described [43].

### Quantification of neutralising antibodies

Blood was collected in serum separation vacutainers (BD Biosciences) and serum was obtained by centrifugation at 524 g for 10 min. The measurement of CSFV neutralising antibody titres in sera was determined using a standard virus neutralisation peroxidise-linked assay using Alfort 187 CSFV and PK-15 cells [41].

### Analysis of IFN-γ production from in vitro stimulated peripheral blood cells

Heparinised blood was collected in heparin vacutainers (BD Biosciences) from pigs every 3 days post-vaccination/challenge. Leukocytes were prepared using a standard protocol. In brief, blood was centrifuged at 800 g for 10 min and visible ‘buffy coat’ material aspirated. Contaminating erythrocytes were lysed by addition of Pharmlyse Buffer (BD Biosciences) and leukocytes incubated for 10 min at RT before being washed three times in Hank's buffered salt solution (HBSS) (Invitrogen). Cells were finally resuspended in RPMI-1640 medium supplemented with 10% foetal calf serum (FCS) and antibiotics (all from Invitrogen). Cell densities were determined by flow cytometry, adjusted to 5×10^6^ cells/ml and 100 µl transferred to wells of a 96 well round-bottom plate. Cells were stimulated by the addition of an equal volume of medium containing C-strain CSFV at a multiplicity of infection (MOI) of 1. For negative control samples, a mock inoculum, prepared from an uninfected PK-15 cell lysate, was added in an equivalent volume. Cells were incubated for 72 hours at 37°C in a humidified 5% CO_2_ atmosphere, then resuspended by repeated pipetting and centrifuged at 400× g for 5 min. Cell free supernatants were removed and immediately stored at −80°C until analysis. IFN-γ was measured in the culture supernatants, diluted 1∶2 in standard diluent buffer using a swine IFN-γ ELISA according to the manufacturer's instructions (Biosource, Invitrogen) and absorbance at 440 nm read using a FLUOstar OPTIMA microplate reader (BMG Labtech, UK).

### Statistical analysis

ANOVA was used for the analysis of fixed effects on different traits using GraphPad Prism 5 (Prism 5 for Windows, Version 5.01, GraphPad Software, Inc. La Jolla, USA). A plot of the total [log10] viraemia versus virus-specific IFN-γ or neutralising antibody responses suggested negative exponential relationships between them, and thus a linear regression was carried out with log total viraemia as the dependent variable and the total virus-specific IFN-γ or neutralising antibody responses as the independent variables. Significant differences in pathological parameters between the vaccinated and unvaccinated animals were analysed with Mann-Whitney-U test.

## Results

### Vaccination five days prior to challenge protected animals against clinical disease and prevented infection of in-contact animals, for both challenge strains

**Figure 1:**
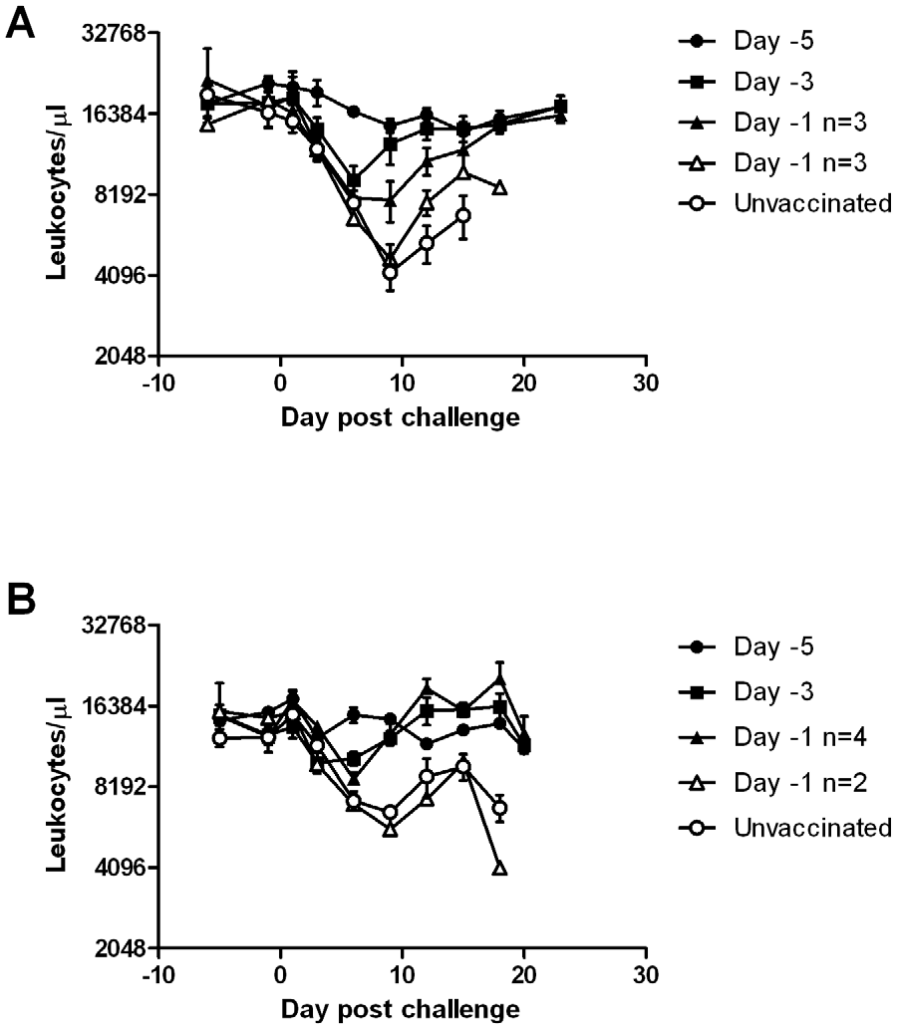
Vaccine induced protection against leukopenia. Leucokyte numbers remained at pre-vaccination levels throughout the experiment in animals vaccinated five days prior to challenge with A) UK2000/7.1 or B) CBR/93 (filled circles), whereas a significant (P<0.05) decrease occurred rapidly after challenge in unvaccinated animals (open circles) and animals vaccinated one day before challenge that developed clinical signs and were euthanized prior to the end of the experiment (open triangles). A smaller, non significant, decrease in leukocyte numbers was observed in animals vaccinated one day prior to challenge that did not develop clinical signs (filled triangles) and those vaccinated three days prior to challenge (filled squares). n = number of animals in groups vaccinated on day prior to challenge that either developed signs and were euthanized or remained clinically healthy. Data are mean leukocyte counts for each group of pigs. Error bars represent SEM.

**Figure 2:**
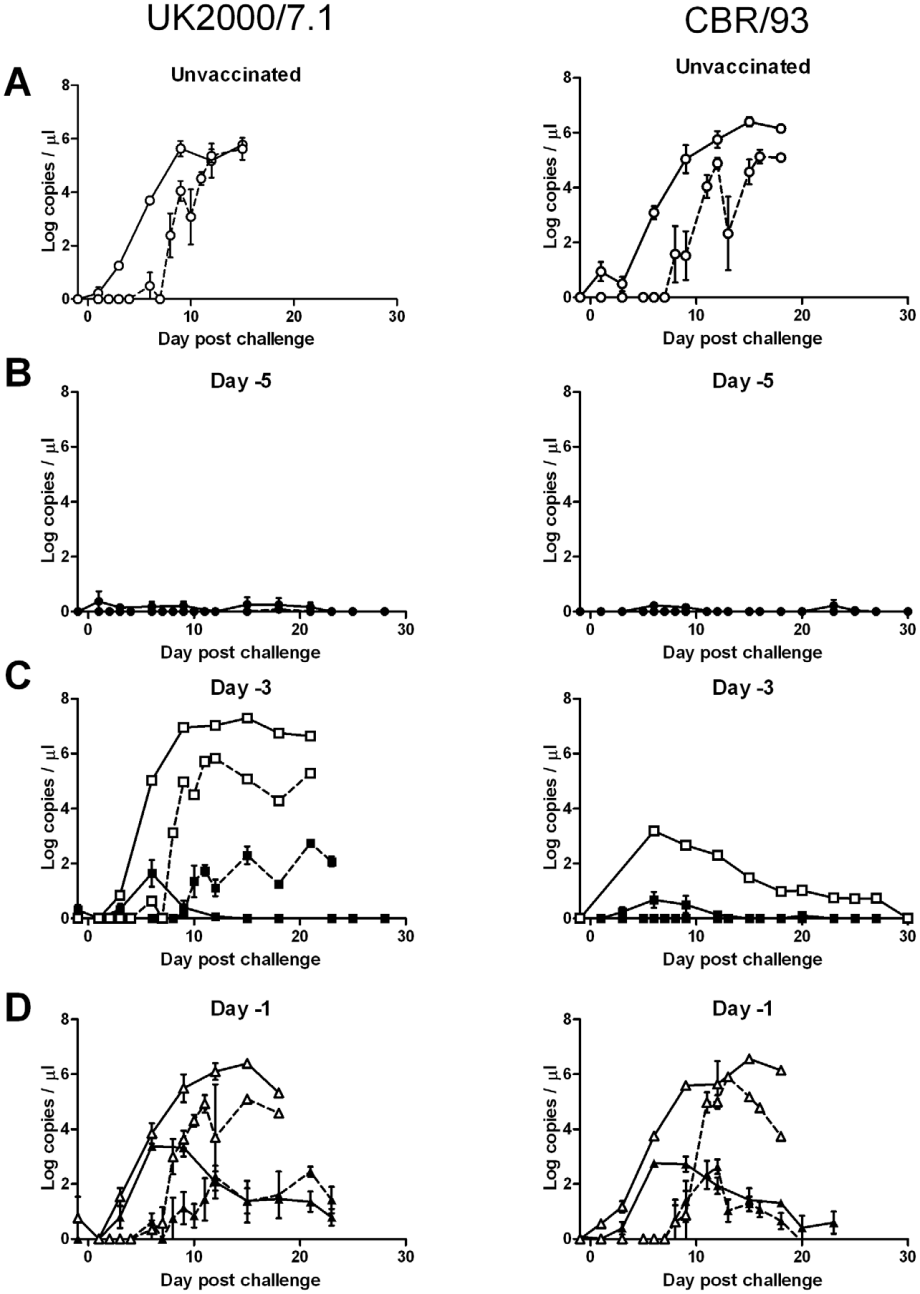
Kinetics of vaccine induced prevention or reduction of viral RNA in blood and nasal secretions. Viral RNA concentrations, determined by real time RT-PCR, in blood (solid lines) and nasal swab samples (dashed lines). A) High levels of viral RNA were detected in blood soon after challenge in unvaccinated animals (open circles), with nasal secretions becoming RNA positive 5–6 days later. B) No viral RNA, or very low levels in the inconclusive range of the assay, was detected in blood or nasal secretion of animals vaccinated 5 days prior to challenge (filled circles). C) High levels of viral RNA was detected in blood and nasal swab samples of one of the animals vaccinated 3 days prior to challenge with UK2000/7.1. An intermediate level of viral RNA was present in blood of one animal vaccinated 3 days prior to challenge with CBR/93 (open squares), whereas the remaining animals in these groups in both experiments (filled squares) had either a transient low level, or no, viral RNA in blood, and either no or intermediate levels of RNA in nasal secretions. D) Animals vaccinated one day prior to challenge either developed clinical disease and were euthanized by 18 dpc (open triangles) or remained healthy for the duration of the experiment (filled triangles).

Unvaccinated animals inoculated with either the genotype 2.1 (UK2000/7.1) or genotype 3 (CBR/93) challenge viruses had similar parameters of infection. Initial clinical signs were observed between 6 to 9 days post challenge (dpc) (Table 1), which coincided with the onset of leukopenia (Fig. 1), thrombocytopenia (data not shown) and detection of high levels of RNA in the blood (Fig. 2A). Clinical signs then increased and animals were euthanized between 14 and 19 dpc for welfare reasons. Post mortem examination confirmed pathological changes consistent with CSF (Table 2). In both experiments, animals placed in-contact with the unvaccinated animals became infected, with clinical signs initially being observed at 15 dpc, 7 days after viral RNA was detected in nasal swab samples from the inoculated animals (Fig. 2A). In-contact animals had haematology and pathology parameters (Table 2) consistent with CSF and infection was confirmed by detection of CSFV RNA in blood. Vaccination of animals five days prior to challenge with either virus prevented all clinical signs of disease. Leukocyte and platelet numbers remained normal, viral RNA was not detected above the inconclusive range of the assay in blood or nasal secretions (Fig. 2B), minimal pathological lesions were observed and the in-contact animals did not develop any trace of infection, indicating that vaccination provided solid protection against clinical disease and prevented subsequent dissemination of these heterogeneous challenge strains.

**Table 1:** Clinical parameters.

Challenge	Experimental	Mean clinical score(a) at days post challenge									Requiring	Infection
virus	group										Euthanasia(b)	of in-contacts(c)
		−6 to −4	−3 to −1	0 to 2	3 to 5	6 to 8	9 to 11	12 to 14	15 to 17	18 to 20		
UK2000	unvaccinated	0 (1)	0 (0)	0 (1)	1 (1)	3 (2)	4 (2)	5 (2)	5 (1)		4/4	Yes
7.1	Day -5	0 (0)	0 (0)	0 (0)	0 (0)	0 (0)	0 (0)	0 (0)	0 (0)	0 (1)	0/6	No
	Day -3	0 (0)	0 (0)	0 (0)	2 (5)	1 (3)	2 (2)	1 (2)	1 (2)	0 (1)	1/6	Yes
	Day -1	0 (0)	0 (0)	0 (1)	0 (0)	1 (1)	2 (2)	3 (2)	2 (1)	1 (2)	3/6	Yes
CBR/93	unvaccinated	0 (0)	0 (0)	0 (1)	0 (1)	1 (2)	3 (2)	3 (2)	4 (2)	4 (2)	4/4	Yes
	Day -5	0 (0)	0 (0)	0 (1)	0 (0)	0 (0)	0 (0)	0 (0)	0 (0)	0 (0)	0/6	No
	Day -3	0 (0)	0 (0)	0 (0)	0 (0)	0 (0)	0 (0)	0 (0)	0 (0)	0 (0)	0/6	No
	Day -1	0 (0)	0 (0)	0 (0)	0 (0)	0 (0)	1 (2)	2 (2)	2 (1)	2 (2)	2/6	Yes

a) Mean clinical scores over 3 day period. Bracketed numbers indicate the standard deviation.

b) Number animals that developed clinical signs and were euthanized before the end of the experiment.

c) In contact animals were identified as infected by detection of CSFV RNA in blood.

**Table 2:** Pathological parameters.

Challenge	Experimental	Euthanized	Pathological score			
virus	group	early(a)				
			Vaccinated+Challenged		In-contact	
			Macro-	Histo-	Macro-	Histo-
	Unvaccinated		29 (4.1)	64 (7.6)	21 (4.2)	51 (0.7)
UK2000/	Day -5		11 (2.2)*	11 (2.3)*	8 (2.1)	14 (2.1)
7.1	Day -3		16 (3.5)*	32 (8.2)*	26 (2.0)	43 (7.6)
	Day -1	No n = 3	14 (4.5)	29 (7.2)	25 (1.5)	51 (12.9)
		Yes n = 3	31 (8.5)	59 (13.9)		
CBR/93	Unvaccinated		31 (5.1)	59 (8.6)	29 (7.1)	55 (11.3)
	Day -5		8 (3.7)*	9 (3.9)*	7 (0.0)	11 (6.9)
	Day -3		8 (1.8)*	15 (4.2)*	5 (1.2)	14 (4.6)
	Day -1	No n = 4	16 (6.4)*	32(13)*	18 (2.0)	34 (5.3)
		Yes n = 2	40 (11)	73 (11)		

a) Number of animals that developed clinical signs and were euthanized early or had no or few signs and survived until the end of the experiment.

*Significant difference to unvaccinated control group (P<0.05).

### Vaccination at very short time points prior to challenge confers some, but not complete, protection against clinical disease and virus dissemination

In the UK2000/7.I challenge experiment one of the animals vaccinated 3 days prior to challenge developed clinical signs soon after challenge which were not considered to be directly due to CSFV. This animal, which was treated with and responded to antibiotics, had high levels of CSFV RNA in both blood and nasal swabs (Fig. 2C) and post mortem examination revealed multifocal bronchopneumonia. The other vaccinated and challenged animals in this group did not manifest any overt clinical signs, although a slight decrease in leukocyte counts was noted in the first week post challenge (Fig. 1), which coincided with a low, transient level of viral RNA in the blood. Low levels of viral RNA were detected in nasal swab samples of these five animals, however this was only subsequent to high levels of virus were being secreted by the antibiotic-treated animal (Fig. 2C). Viral RNA was detected in the blood of the three in-contact animals in this group from dpc 12, they began to develop obvious signs at dpc19 and were euthanized between dpc 19 and 22. In contrast, for the genotype 3.3 (CBR/93) strain none of the animals vaccinated 3 days prior to challenge had any notable clinical changes or decrease in leukocytes numbers (Fig. 1). One of the vaccinated animals in this group had intermediate levels of viral RNA in the blood from 6 dpc which then decreased (Fig. 2C). However, no viral RNA was detected in nasal swab samples from any animals in this group and the in-contact animals in this group did not become infected.

In the groups vaccinated 1 day before challenge, with either of the challenge strains, some of the vaccinated animals had clinical signs from 7–10 dpc and were euthanized for welfare reasons, whereas the other vaccinated animals remained apparently healthy for the duration of the experiment (Table 1). The haematological, pathological and virological data clearly delineated two different outcomes of challenge at this short period post-vaccination (Figure 1, 2D, Table 2). The animals that developed clinical signs and were euthanized early were not protected by the vaccine, with all parameters indicating a disease resembling the unvaccinated control group. The remaining animals were infected by the challenge virus but were able to control the infection; haematology returned to normal, viral RNA concentrations in the blood decreased and only low levels of viral RNA were detected in nasal secretions. Despite this evidence of a degree of clinical protection among the vaccinates, all in-contact animals in both day −1 vaccination groups developed CSF, indicating that transmission was not prevented when animals were challenged 24 hours after vaccination.

### Virus-specific T cell IFN-γ responses correlate with complete protection induced by vaccination 5 days before challenge whereas both neutralising antibody and T cell IFN-γ responses contribute to protection at later time points

**Figure 3:**
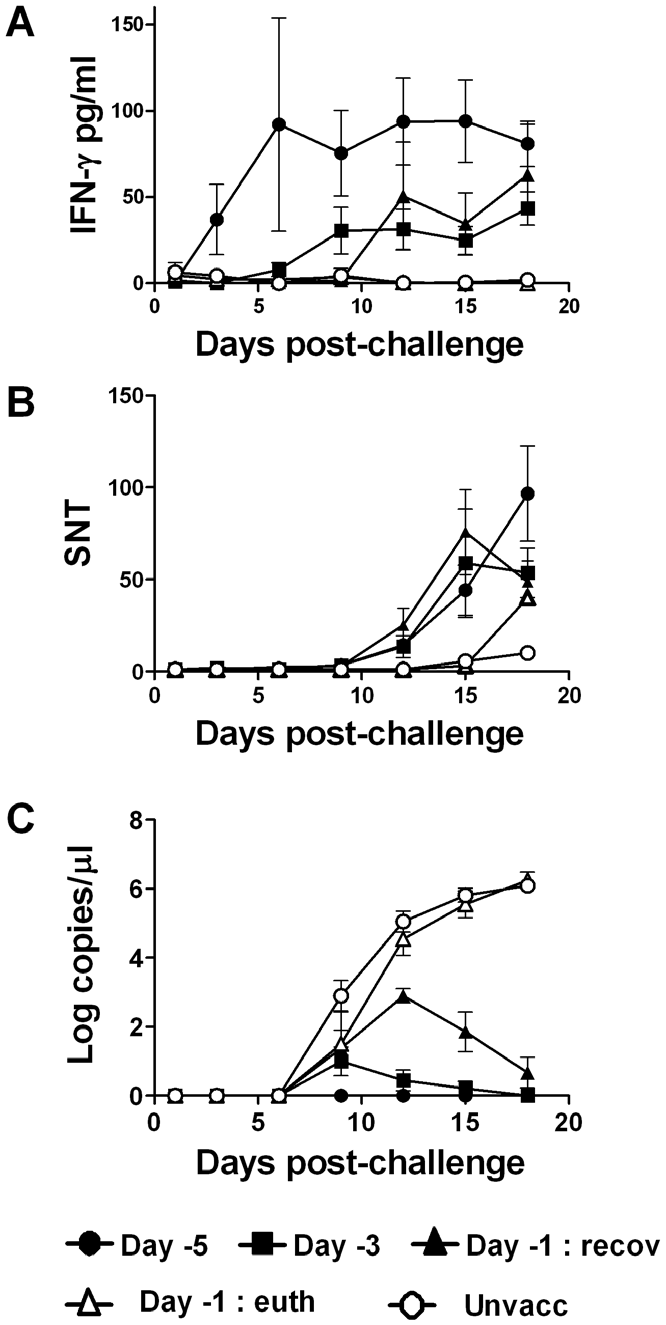
Association of virus-specific IFN-γ and serum neutralising antibody responses with viraemia. Pigs were vaccinated with C-strain CSFV on days -5, -3 or -1 and were then challenged, together with groups of unvaccinated pigs, with UK2000/7.1 or CBR93 CSFV isolates. According to clinical outcome following challenge, the day -1 vaccinated pigs were separated into two groups; (recov) animals that experienced mild signs followed by recovery and (euth) animals that developed severe signs necessitating euthanasia. (A) Peripheral blood leukocyte (PBL) IFN-γ responses were measured by ELISA following *in vitro* stimulation with C-strain CSFV or an uninfected cell preparation (Mock) and data presented as the mock corrected values. (B) Serum neutralisation titres (SNT) against the CSFV reference strain Alfort 187 were assessed *in vitro*. (C) Viraemia in was measured by qRT-PCR and expressed as log_10_ viral RNA copies/µl blood. Data from the UK2000/7.1 and CBR/93 experiments were combined and results expressed as the mean data for each group of pigs and error bars represent SEM.

Since both neutralising antibody and virus-specific T cell responses may exert inhibitory effects on CSFV, we investigated the kinetics of these responses and compared this to the course of viraemia observed following vaccination and challenge (Fig. 3). Virus-specific T cell IFN-γ responses for both challenge strains were measured longitudinally following *in vitro* stimulation of peripheral blood leukocytes with C-strain CSFV (Fig. 3A). Of the challenge time-points studied CSFV specific IFN-γ T cell responses were strongest in the animals vaccinated 5 days before challenge, which peaked at 6 dpc (11 days post-vaccination) and remained elevated (p<0.05). For all the day -3 vaccinates, and the day -1 vaccinates that controlled the infection, virus specific IFN-γ responses were detected from day 9 and 12 post-challenge, respectively, and were significantly reduced compared to the day -5 vaccinates (p<0.05). No virus specific IFN-γ responses were detected from any of the day-1 vaccinated animals that succumbed to the challenge infection or from the unvaccinated challenge control pigs. In contrast, virus neutralising antibody titres were detected from day 12 post-challenge in the groups vaccinated on day -5, -3 and the day -1 vaccinates that recovered from the challenge infection (Fig. 3B). Neutralising antibody was only detected from day 15 post-challenge in the day -1 vaccinates that succumbed to infection and the challenge controls. The significance of the responses became further apparent when they were correlated with the viraemia detected in these animals (Fig. 3C). A log-linear regression analysis showed a negative exponential relationship between the total [log10] viraemia and both the virus-specific IFN-γ and neutralising antibody responses (p<0.05). The day -5 vaccinates had robust IFN-γ responses, and antibody appeared later in the absence of a detectable viraemia, whereas the day -1 and unvaccinated animals that succumbed to challenge had undetectable IFN-γ responses, delayed antibody responses and uncontrolled viraemia. Interestingly, the decline and disappearance of viraemia in the day -3 vaccinates and the day -1 vaccinates that controlled the challenge infection, occurred coincidently with the appearance of virus-specific IFN-γ responses and neutralising antibody.

## Discussion

C-strain vaccines, such as the Riemser® vaccine, are known to be highly efficacious at preventing clinical disease. However, despite their use in the field for many years, few standard vaccination experiments have investigated the protection afforded against diverse genotype 3 viruses, and the ability of vaccination to prevent infection of naive in-contact animals is often not considered. Genotype 3 viruses are less common than the other CSFV genotypes and have, thus far, largely been restricted to Asia: A distinct clade of genotype 3.2 viruses were isolated in Korea between 1988 and 1999 [44], 18 genotype 3.3 viruses were isolated in Thailand between 1988 and 1996 [30] and genotype 3.4 viruses have been found in Japan [45] and were endemic in Taiwan until 1996 [46], [47]. Prophylactic vaccination, for example with LPC and GPE^−^ vaccines [45], [46], has reduced the incidence of CSF in these regions and group 3 viruses have not been reported recently. However, the diversity of the viruses within this group implies that they have been circulating for some time and the possibility of these viruses re-emerging, for example from wild suidae, remains. This study provides conclusive evidence that the Riemser® vaccine, which is a tissue-culture adapted version of C-strain, provides an equivalent and complete protection by 5 days post-vaccination against disease and spread of genotype 3 and genotype 2 viruses.

As well as being a representative of genotype 3, the CBR/93 strain is of additional interest as it is one of only two known isolates that do not bind to monoclonal antibodies against the highly conserved, immunodominant TAVSPTLLR epitope [32]. Although we observed no difference in the rapid vaccine protection afforded against this strain and the genotype 2.1 isolate, the efficacy of long term protection, when the role of neutralising antibodies is likely to be of greater importance, still needs to be assessed.

The extent of protection, particularly against virus transmission, induced by C-strain vaccination at times earlier than 5 days post-vaccination has not been described in detail. For an emergency vaccination, the rapidity with which an intervention will prevent spread of virus is of utmost importance. This knowledge is crucial for making decisions on use of emergency vaccination strategies compared to a pre-emptive slaughter policy. These investigations indicate that by 3 days post-vaccination, the majority of animals were substantially protected from clinical disease and viraemia and the amount of virus present in nasal secretions was markedly reduced. The difference between the fates of the in contact animals in the groups vaccinated 3 days prior to challenge in the two experiments was, almost certainly, due to the presence of one animal with underlying health issues in the UK2000/7.1 challenge experiment that was unable to control the disease. This animal had a high level of virus in nasal secretions that was sufficient to result in infection of the in-contact animals. The lower level of virus present in nasal swabs of other animals in this pen, at a time when they were no longer viraemic, is likely to originate from environmental contamination derived from this one animal rather than secretion from the other animals themselves. Interestingly, although one animal vaccinated 3 days prior to challenge with CBR/93 had an intermediate level of virus in the blood, vaccination provided sufficient protection to prevent nasal secretion and infection of the in-contact animals. Even as early as one day post-vaccination a proportion of the animals were protected to some degree. Although, this was insufficient to prevent infection of animals in direct contact, there was a reduction in the overall level of virus excreted into the environment. As virus transmission is affected by the amount of virus excreted [48], these results indicate that vaccination would reduce the potential for virus to spread between farms from as early as one day post-vaccination.

The disadvantage of current live attenuated vaccines for CSF is the inability to distinguish vaccinated and infected animals by serology, leading to the potential for field virus to hide within a vaccinated population. The vaccinated animals in this study that became infected were either not protected, with viral and immunological parameters identical to unvaccinated animals, or were protected to a certain extent. The majority of infected animals with no protection would likely be clinically apparent and, as only very low levels of vaccine virus are found in blood after intramuscular vaccination [49], a high level of virus in blood would be indicative of field infection. Such infections could subsequently be easily distinguished by genetic-based differential tests, such as sequencing or discriminatory PCRs, which have been applied successfully during oral vaccination campaigns in wild boar [50]. Those animals that are partially protected and harbour lower levels of virus would be more difficult to identify, but strategies such as inclusion of unvaccinated sentinel animals within a vaccinated herd, in combination with discriminatory PCR assays, could be applied as part of an emergency vaccination and testing regime, so as to substantially reduce the risk that the field virus would remain undetected. As highlighted by the animal with a low level viraemia, which did not transmit virus to animals in direct contact, these partially protected animals would be of minimal risk of further disease transmission [14]. However, the risk posed by the level of virus present in meat and products from such vaccinated and infected animals, if it were allowed to be traded, remains a question that deserves further research.

It has previously been reported that CSFV C-strain can protect pigs 7 days post vaccination, which precedes the appearance of virus-neutralising serum antibodies, but not T cell responses [1], [34], and animals protected from challenge by vaccination with an E2 based DNA vaccine have increased levels of CSFV specific IFN-γ producing cells compared to unvaccinated controls [51]. However, the temporally associated stratification of protection in the vaccination study we present here has, for the first time, convincingly shown a correlation between the induction of T cell responses and the protection afforded. Whilst it remains to be determined if this correlation represents a direct causal interaction of IFN-γ response with CSFV, it highlights that efforts toward future generation CSFV vaccines, particularly those that would be used for emergency strategies, should consider this a critical correlate of protection.

In conclusion these data have addressed important questions about how this vaccine might perform if applied in an emergency vaccination strategy. As well as demonstrating broad protection against diverse genotypes these studies demonstrate how rapidly a reduction in between-herd transmission could be achieved.
